# (4-Chloro­acetanilido-κ^2^ *N*,*O*)bis­[2-(pyridin-2-yl)phenyl-κ^2^ *C* ^1^,*N*]iridium(III)

**DOI:** 10.1107/S1600536813000433

**Published:** 2013-01-12

**Authors:** Lijun Sun, Songlin Zhang, Qijun Song

**Affiliations:** a School of Chemical and Material Engineering, Jiangnan University, Wuxi 214122, Jiangsu Province, People’s Republic of China

## Abstract

In the neutral mononuclear iridium(III) title compound, [Ir(C_8_H_7_ClNO)(C_11_H_8_N)_2_], the Ir^III^ atom adopts an octa­hedral geometry, and is coordinated by two 2-phenyl­pyridyl ligands and one anionic 4-chloro­acetanilide ligand. The 2-phenyl­pyridyl ligands are arranged in a *cis*-*C*,*C*′ and *cis*-*N*,*N*′ fashion. Each 2-phenyl­pyridyl ligand forms a five-membered ring with the Ir^III^ atom. The 2-phenyl­pyridyl planes are perpendicular to each other [dihedral angle = 89.9 (1)°]. The Ir—C and Ir—N bond lengths are comparable to those reported for related iridium(III) 2-phenyl­pyridyl complexes. The remaining two coordination sites are occupied by the amidate N and O atoms, which form a four-membered ring with the iridium atom (Ir—N—C—O). The amidate plane is nearly perpendicular to both 2-phenyl­pyridyl ligands [dihedral angles = 87.8 (2) and 88.3 (2)°].

## Related literature

For related iridium(III) complexes containing 2-phenyl­pyridyl derivatives as cyclo­metalating ligands, see: Lamansky *et al.* (2001 ▶); Tamayo *et al.* (2003 ▶); Yang *et al.* (2011 ▶); You & Park (2005 ▶); Zhang *et al.* (2011 ▶). For the coordination geometry of some heteroleptic iridium(III) complexes containing amidate ancillary ligands, see: Yang *et al.* (2011 ▶); Zhang *et al.* (2011 ▶). For a general procedure for the preparation of a chloride-bridged iridium(III) dimer, see: Nonoyama (1974 ▶).
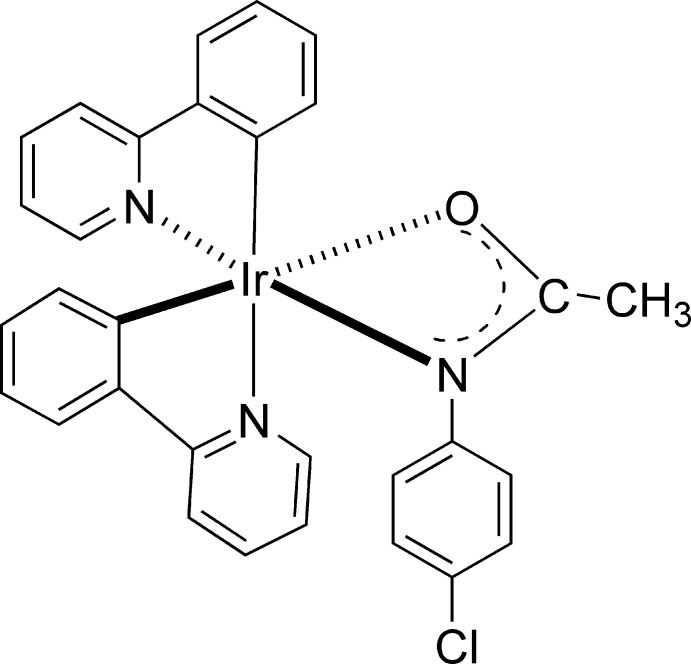


## Experimental

### 

#### Crystal data

[Ir(C_8_H_7_ClNO)(C_11_H_8_N)_2_]
*M* *_r_* = 669.16
Orthorhombic, 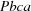

*a* = 12.8391 (15) Å
*b* = 11.0697 (13) Å
*c* = 35.897 (4) Å
*V* = 5101.8 (11) Å^3^
*Z* = 8
Mo *K*α radiation
μ = 5.37 mm^−1^
*T* = 293 K
0.58 × 0.20 × 0.20 mm

#### Data collection

Rigaku Mercury diffractometer
Absorption correction: multi-scan (*REQAB*; Jacobson, 1998 ▶) *T*_min_ = 0.073, *T*_max_ = 0.584
41875 measured reflections
4668 independent reflections
4140 reflections with *I* > 2σ(*I*)
*R*_int_ = 0.064

#### Refinement

*R*[*F* ^2^ > 2σ(*F* ^2^)] = 0.058
*wR*(*F* ^2^) = 0.099
*S* = 1.11
4668 reflections
326 parameters
H-atom parameters constrained
Δρ_max_ = 1.56 e Å^−3^
Δρ_min_ = −0.76 e Å^−3^

### 

Data collection: *CrystalClear* (Rigaku, 2007 ▶); cell refinement: *CrystalClear*; data reduction: *CrystalClear*; program(s) used to solve structure: *SHELXS97* (Sheldrick, 2008 ▶); program(s) used to refine structure: *SHELXL97* (Sheldrick, 2008 ▶); molecular graphics: *SHELXTL* (Sheldrick, 2008 ▶); software used to prepare material for publication: *SHELXTL*.

## Supplementary Material

Crystal structure: contains datablock(s) I, global. DOI: 10.1107/S1600536813000433/pk2461sup1.cif

Structure factors: contains datablock(s) I. DOI: 10.1107/S1600536813000433/pk2461Isup2.hkl

Additional supplementary materials:  crystallographic information; 3D view; checkCIF report

## Figures and Tables

**Fig. 1.**
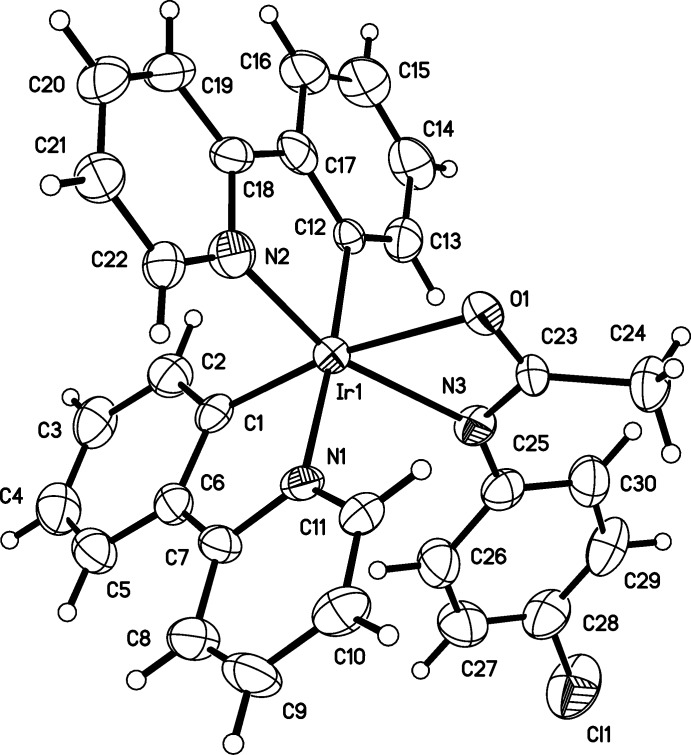
Molecular structure of the title compound with ellipsoids drawn at the 50% probability level.

## supplementary crystallographic information

### Comment

Previously, the synthesis, characterization and photophysical properties of a series of iridium(III) complexes have been reported, which contain 2-phenylpyridine and their derivatives as cyclometalating ligands and various monoanionic ligands as the ancillary ligand, such as acetylacetone, picolinate, amidate and others (Lamansky *et al.*, 2001; Tamayo *et al.*, 2003; Yang *et al.*, 2011; You *et al.*, 2005; Zhang *et al.*, 2011). These complexes have shown good photoluminescence. The remote substituent effect of amidate ligand on photophysical properties was recognized (Zhang *et al.*, 2011). The simple and efficient fine tuning of the emission properties of iridium(III) amidate complexes can potentially be achieved *via* the alternation of the subtle electronic effects of amidate ancillary ligands. Herein, an acetanilide ligand was used as the ancillary ligand, which contains a *para*-chlorine on the *N*-phenyl ring. The crystal structure of the resulting phenylpyridyl iridium(III) amidate complex was obtained. The iridium(III) center adopts an octahedral geometry, which is coordinated by two 2-phenylpyridyl ligands and one *para*-chloroacetanilide ancillary ligand. The two 2-phenylpyridyl ligands are arranged in a *cis*-C, C' and *cis*-N, N' fashion, whose planes are nearly perpendicular to each other.

### Experimental

The dichloro-bridged dimeric complex [(ppy)_2_IrCl]_2_ was obtained by reaction of IrCl_3_ with ppy ligand according to a general procedure originally developed by Nonoyama *et al.* (1974). Into a 100 ml Schlenk tube were added the dichloro-bridged dimer, 2.5 equiv. of amide ligand and 10 equiv. of sodium methoxide in 15 ml CH_2_Cl_2_ solvent under dinitrogen atmosphere. The mixture was stirred at room temperature for 48 h. The product mixture was filtered to remove the solids. The solvent of the resulting filtrate was removed by rotational evaporation to give the crude product powder. The powder was washed sequentially with *n*-hexane and ethers. Recrystallization by evaporation of a soution in CH_2_Cl_2_/*n*-hexane (in a 1:1 ratio) mixed solvent gave the final crystalline product.

### Refinement

All H atoms were placed in geometrically idealized positions and constrained to ride on their parent atoms with C–H of 0.93–0.96 Å, and *U*_iso_(H) = 1.2–1.5 *U*_eq_ (C).

### Crystal data

**Table:**

[Ir(C_8_H_7_ClNO)(C_11_H_8_N)_2_]	*F*(000) = 2608
*M**_r_* = 669.16	*D*_x_ = 1.742 Mg m^−^^3^
Orthorhombic, *P**b**c**a*	Mo *K*α radiation, λ = 0.71070 Å
Hall symbol: -P 2ac 2ab	Cell parameters from 15543 reflections
*a* = 12.8391 (15) Å	θ = 3.2–25.3°
*b* = 11.0697 (13) Å	µ = 5.37 mm^−^^1^
*c* = 35.897 (4) Å	*T* = 293 K
*V* = 5101.8 (11) Å^3^	Block, yellow
*Z* = 8	0.58 × 0.20 × 0.20 mm

### Data collection

**Table:**

Rigaku Mercury diffractometer	4668 independent reflections
Radiation source: fine-focus sealed tube	4140 reflections with *I* > 2σ(*I*)
Graphite monochromator	*R*_int_ = 0.064
Detector resolution: 7.31 pixels mm^-1^	θ_max_ = 25.4°, θ_min_ = 3.2°
ω scans	*h* = −15→15
Absorption correction: multi-scan (*REQAB*; Jacobson, 1998)	*k* = −12→13
*T*_min_ = 0.073, *T*_max_ = 0.584	*l* = −39→43
41875 measured reflections	

### Refinement

**Table:**

Refinement on *F*^2^	Primary atom site location: structure-invariant direct methods
Least-squares matrix: full	Secondary atom site location: difference Fourier map
*R*[*F*^2^ > 2σ(*F*^2^)] = 0.058	Hydrogen site location: inferred from neighbouring sites
*wR*(*F*^2^) = 0.099	H-atom parameters constrained
*S* = 1.11	*w* = 1/[σ^2^(*F*_o_^2^) + (0.0063*P*)^2^ + 60.1899*P*] where *P* = (*F*_o_^2^ + 2*F*_c_^2^)/3
4668 reflections	(Δ/σ)_max_ = 0.002
326 parameters	Δρ_max_ = 1.56 e Å^−^^3^
0 restraints	Δρ_min_ = −0.76 e Å^−^^3^

### Special details

**Table:**

Geometry. All e.s.d.'s (except the e.s.d. in the dihedral angle between two l.s. planes) are estimated using the full covariance matrix. The cell e.s.d.'s are taken into account individually in the estimation of e.s.d.'s in distances, angles and torsion angles; correlations between e.s.d.'s in cell parameters are only used when they are defined by crystal symmetry. An approximate (isotropic) treatment of cell e.s.d.'s is used for estimating e.s.d.'s involving l.s. planes.
Refinement. Refinement of *F*^2^ against ALL reflections. The weighted *R*-factor *wR* and goodness of fit *S* are based on *F*^2^, conventional *R*-factors *R* are based on *F*, with *F* set to zero for negative *F*^2^. The threshold expression of *F*^2^ > σ(*F*^2^) is used only for calculating *R*-factors(gt) *etc*. and is not relevant to the choice of reflections for refinement. *R*-factors based on *F*^2^ are statistically about twice as large as those based on *F*, and *R*- factors based on ALL data will be even larger.

### Fractional atomic coordinates and isotropic or equivalent isotropic displacement parameters (Å^2^)

**Table:**

	*x*	*y*	*z*	*U*_iso_*/*U*_eq_	
Ir1	0.52201 (3)	0.20795 (3)	0.647807 (9)	0.03746 (11)	
Cl1	0.3095 (3)	0.6436 (3)	0.50134 (10)	0.1069 (13)	
O1	0.5741 (5)	0.3599 (5)	0.68362 (16)	0.0476 (15)	
N1	0.6555 (5)	0.2045 (6)	0.61709 (18)	0.0394 (16)	
N2	0.5537 (7)	0.0664 (7)	0.6804 (2)	0.061 (2)	
N3	0.4950 (5)	0.3970 (6)	0.63019 (19)	0.0417 (18)	
C1	0.4800 (7)	0.0911 (7)	0.6084 (2)	0.039 (2)	
C2	0.3862 (8)	0.0282 (9)	0.6052 (3)	0.055 (3)	
H2	0.3350	0.0393	0.6231	0.066*	
C3	0.3673 (9)	−0.0505 (9)	0.5759 (3)	0.064 (3)	
H3	0.3040	−0.0910	0.5741	0.076*	
C4	0.4440 (10)	−0.0680 (10)	0.5492 (3)	0.067 (3)	
H4	0.4316	−0.1196	0.5293	0.081*	
C5	0.5373 (9)	−0.0104 (9)	0.5519 (3)	0.059 (3)	
H5	0.5885	−0.0240	0.5340	0.070*	
C6	0.5566 (8)	0.0689 (8)	0.5813 (2)	0.045 (2)	
C7	0.6552 (7)	0.1308 (8)	0.5868 (2)	0.044 (2)	
C8	0.7431 (9)	0.1250 (10)	0.5643 (3)	0.062 (3)	
H8	0.7437	0.0739	0.5438	0.075*	
C9	0.8285 (9)	0.1937 (11)	0.5722 (3)	0.067 (3)	
H9	0.8873	0.1894	0.5571	0.080*	
C10	0.8271 (8)	0.2694 (10)	0.6026 (3)	0.061 (3)	
H10	0.8841	0.3180	0.6083	0.074*	
C11	0.7394 (7)	0.2712 (8)	0.6244 (3)	0.048 (2)	
H11	0.7384	0.3212	0.6452	0.057*	
C12	0.3849 (5)	0.2006 (7)	0.6768 (2)	0.0288 (16)	
C13	0.3043 (8)	0.2713 (9)	0.6708 (3)	0.056 (3)	
H13	0.3085	0.3286	0.6519	0.067*	
C14	0.2129 (8)	0.2639 (11)	0.6915 (3)	0.066 (3)	
H14	0.1575	0.3160	0.6869	0.079*	
C15	0.2069 (9)	0.1791 (11)	0.7185 (4)	0.073 (3)	
H15	0.1470	0.1727	0.7329	0.088*	
C16	0.2895 (8)	0.1019 (10)	0.7247 (3)	0.064 (3)	
H16	0.2853	0.0423	0.7429	0.077*	
C17	0.3792 (7)	0.1150 (9)	0.7032 (3)	0.049 (2)	
C18	0.4726 (8)	0.0400 (8)	0.7067 (2)	0.046 (2)	
C19	0.4866 (8)	−0.0518 (9)	0.7327 (2)	0.055 (3)	
H19	0.4337	−0.0694	0.7495	0.066*	
C20	0.5770 (9)	−0.1162 (10)	0.7337 (3)	0.063 (3)	
H20	0.5858	−0.1765	0.7515	0.076*	
C21	0.6547 (9)	−0.0930 (9)	0.7087 (3)	0.059 (3)	
H21	0.7158	−0.1378	0.7096	0.071*	
C22	0.6436 (7)	−0.0036 (8)	0.6822 (3)	0.047 (2)	
H22	0.6970	0.0099	0.6652	0.056*	
C23	0.5404 (7)	0.4395 (8)	0.6608 (2)	0.042 (2)	
C24	0.5570 (8)	0.5710 (8)	0.6703 (3)	0.060 (3)	
H24A	0.4970	0.6015	0.6832	0.091*	
H24B	0.5673	0.6163	0.6478	0.091*	
H24C	0.6173	0.5789	0.6859	0.091*	
C25	0.4504 (7)	0.4570 (9)	0.6010 (3)	0.050 (2)	
C26	0.4552 (10)	0.4051 (10)	0.5657 (3)	0.069 (3)	
H26	0.4883	0.3310	0.5626	0.083*	
C27	0.4117 (9)	0.4619 (11)	0.5354 (3)	0.071 (3)	
H27	0.4153	0.4259	0.5121	0.085*	
C28	0.3634 (10)	0.5700 (11)	0.5395 (3)	0.071 (3)	
C29	0.3555 (10)	0.6215 (10)	0.5731 (4)	0.076 (4)	
H29	0.3217	0.6954	0.5754	0.091*	
C30	0.3972 (9)	0.5661 (9)	0.6046 (3)	0.067 (3)	
H30	0.3896	0.6018	0.6279	0.081*	

### Atomic displacement parameters (Å^2^)

**Table:**

	*U*^11^	*U*^22^	*U*^33^	*U*^12^	*U*^13^	*U*^23^
Ir1	0.04144 (19)	0.03439 (18)	0.03655 (19)	0.00003 (16)	0.00421 (15)	−0.00038 (16)
Cl1	0.142 (3)	0.083 (2)	0.095 (3)	−0.004 (2)	−0.052 (2)	0.027 (2)
O1	0.058 (4)	0.045 (4)	0.040 (3)	−0.003 (3)	0.001 (3)	−0.008 (3)
N1	0.043 (4)	0.041 (4)	0.034 (4)	−0.001 (4)	0.006 (3)	0.009 (3)
N2	0.076 (6)	0.054 (5)	0.052 (5)	−0.013 (5)	0.000 (5)	−0.005 (4)
N3	0.044 (4)	0.042 (4)	0.039 (4)	0.010 (3)	−0.006 (3)	0.010 (3)
C1	0.045 (5)	0.034 (5)	0.039 (5)	0.003 (4)	−0.006 (4)	0.011 (4)
C2	0.059 (6)	0.057 (6)	0.049 (6)	−0.003 (5)	−0.006 (5)	0.001 (5)
C3	0.070 (7)	0.051 (6)	0.069 (7)	−0.017 (6)	−0.018 (6)	−0.003 (6)
C4	0.092 (9)	0.057 (7)	0.053 (7)	0.004 (7)	−0.021 (6)	−0.006 (5)
C5	0.072 (8)	0.058 (6)	0.045 (6)	0.012 (6)	0.000 (5)	−0.004 (5)
C6	0.058 (6)	0.039 (5)	0.039 (5)	0.008 (5)	−0.004 (4)	0.004 (4)
C7	0.053 (6)	0.046 (5)	0.032 (5)	0.005 (5)	0.004 (4)	0.000 (4)
C8	0.069 (7)	0.064 (7)	0.054 (6)	0.007 (6)	0.019 (6)	0.006 (6)
C9	0.063 (7)	0.083 (8)	0.054 (6)	0.011 (7)	0.025 (5)	−0.004 (6)
C10	0.048 (6)	0.066 (7)	0.070 (7)	−0.012 (5)	0.007 (5)	0.011 (6)
C11	0.051 (5)	0.041 (5)	0.051 (5)	−0.008 (5)	0.008 (5)	0.006 (4)
C12	0.024 (4)	0.030 (4)	0.033 (4)	0.002 (4)	0.004 (3)	−0.006 (4)
C13	0.060 (6)	0.044 (6)	0.063 (6)	−0.006 (5)	0.009 (5)	−0.006 (5)
C14	0.042 (6)	0.075 (8)	0.082 (8)	0.007 (6)	0.011 (6)	−0.019 (7)
C15	0.048 (6)	0.085 (9)	0.086 (9)	−0.017 (6)	0.025 (6)	−0.011 (7)
C16	0.054 (6)	0.073 (7)	0.066 (7)	−0.019 (6)	0.028 (5)	−0.009 (6)
C17	0.050 (6)	0.051 (6)	0.046 (5)	−0.016 (5)	0.003 (4)	−0.021 (5)
C18	0.055 (6)	0.049 (5)	0.033 (5)	−0.015 (5)	0.004 (4)	−0.003 (4)
C19	0.066 (7)	0.061 (6)	0.037 (5)	−0.018 (6)	0.000 (5)	0.006 (5)
C20	0.083 (8)	0.058 (6)	0.048 (6)	0.004 (6)	−0.013 (6)	0.014 (5)
C21	0.072 (7)	0.057 (6)	0.048 (6)	0.012 (6)	−0.010 (5)	0.004 (5)
C22	0.048 (5)	0.041 (5)	0.051 (6)	0.007 (4)	0.000 (4)	0.002 (4)
C23	0.052 (6)	0.031 (5)	0.043 (5)	−0.007 (4)	0.006 (4)	−0.009 (4)
C24	0.070 (7)	0.043 (6)	0.068 (7)	−0.009 (5)	−0.012 (6)	−0.007 (5)
C25	0.044 (6)	0.050 (6)	0.055 (6)	−0.012 (5)	0.001 (5)	0.003 (5)
C26	0.104 (9)	0.050 (6)	0.055 (7)	0.013 (6)	−0.006 (6)	0.001 (5)
C27	0.092 (9)	0.076 (8)	0.045 (6)	0.008 (7)	−0.009 (6)	0.002 (6)
C28	0.087 (9)	0.065 (8)	0.060 (7)	−0.009 (7)	−0.023 (6)	0.015 (6)
C29	0.083 (9)	0.043 (6)	0.103 (10)	−0.002 (6)	−0.019 (8)	0.014 (7)
C30	0.084 (8)	0.047 (6)	0.072 (8)	0.005 (6)	−0.001 (6)	−0.004 (6)

### Geometric parameters (Å, º)

**Table:**

Ir1—C1	1.990 (9)	C12—C13	1.315 (12)
Ir1—N2	1.998 (9)	C12—C17	1.344 (12)
Ir1—N1	2.039 (7)	C13—C14	1.392 (13)
Ir1—C12	2.045 (7)	C13—H13	0.9300
Ir1—N3	2.213 (7)	C14—C15	1.350 (15)
Ir1—O1	2.220 (6)	C14—H14	0.9300
Ir1—C23	2.616 (8)	C15—C16	1.380 (15)
Cl1—C28	1.738 (11)	C15—H15	0.9300
O1—C23	1.279 (10)	C16—C17	1.395 (12)
N1—C11	1.331 (11)	C16—H16	0.9300
N1—C7	1.359 (11)	C17—C18	1.463 (13)
N2—C22	1.391 (12)	C18—C19	1.390 (12)
N2—C18	1.436 (12)	C19—C20	1.363 (14)
N3—C23	1.330 (10)	C19—H19	0.9300
N3—C25	1.367 (11)	C20—C21	1.365 (14)
C1—C2	1.396 (13)	C20—H20	0.9300
C1—C6	1.407 (12)	C21—C22	1.380 (12)
C2—C3	1.387 (13)	C21—H21	0.9300
C2—H2	0.9300	C22—H22	0.9300
C3—C4	1.387 (15)	C23—C24	1.510 (12)
C3—H3	0.9300	C24—H24A	0.9600
C4—C5	1.360 (15)	C24—H24B	0.9600
C4—H4	0.9300	C24—H24C	0.9600
C5—C6	1.394 (13)	C25—C26	1.391 (13)
C5—H5	0.9300	C25—C30	1.394 (14)
C6—C7	1.453 (13)	C26—C27	1.375 (14)
C7—C8	1.388 (13)	C26—H26	0.9300
C8—C9	1.364 (15)	C27—C28	1.356 (15)
C8—H8	0.9300	C27—H27	0.9300
C9—C10	1.376 (14)	C28—C29	1.336 (16)
C9—H9	0.9300	C29—C30	1.394 (15)
C10—C11	1.371 (13)	C29—H29	0.9300
C10—H10	0.9300	C30—H30	0.9300
C11—H11	0.9300		
			
C1—Ir1—N2	87.8 (3)	C13—C12—C17	119.5 (8)
C1—Ir1—N1	80.3 (3)	C13—C12—Ir1	124.8 (7)
N2—Ir1—N1	97.5 (3)	C17—C12—Ir1	115.7 (6)
C1—Ir1—C12	95.8 (3)	C12—C13—C14	122.8 (10)
N2—Ir1—C12	81.2 (3)	C12—C13—H13	118.6
N1—Ir1—C12	176.0 (3)	C14—C13—H13	118.6
C1—Ir1—N3	111.7 (3)	C15—C14—C13	118.2 (10)
N2—Ir1—N3	160.2 (3)	C15—C14—H14	120.9
N1—Ir1—N3	89.7 (3)	C13—C14—H14	120.9
C12—Ir1—N3	92.7 (3)	C14—C15—C16	120.2 (10)
C1—Ir1—O1	170.1 (3)	C14—C15—H15	119.9
N2—Ir1—O1	101.2 (3)	C16—C15—H15	119.9
N1—Ir1—O1	94.3 (3)	C15—C16—C17	118.7 (11)
C12—Ir1—O1	89.7 (3)	C15—C16—H16	120.7
N3—Ir1—O1	59.8 (2)	C17—C16—H16	120.7
C1—Ir1—C23	142.0 (3)	C12—C17—C16	120.6 (10)
N2—Ir1—C23	130.2 (3)	C12—C17—C18	114.6 (8)
N1—Ir1—C23	92.2 (3)	C16—C17—C18	124.8 (10)
C12—Ir1—C23	91.5 (3)	C19—C18—N2	119.7 (9)
N3—Ir1—C23	30.5 (3)	C19—C18—C17	125.3 (9)
O1—Ir1—C23	29.2 (2)	N2—C18—C17	115.0 (8)
C23—O1—Ir1	92.8 (5)	C20—C19—C18	120.7 (10)
C11—N1—C7	119.5 (8)	C20—C19—H19	119.7
C11—N1—Ir1	124.2 (6)	C18—C19—H19	119.7
C7—N1—Ir1	116.2 (6)	C19—C20—C21	120.4 (10)
C22—N2—C18	117.2 (8)	C19—C20—H20	119.8
C22—N2—Ir1	129.3 (7)	C21—C20—H20	119.8
C18—N2—Ir1	113.4 (7)	C20—C21—C22	120.8 (10)
C23—N3—C25	130.2 (8)	C20—C21—H21	119.6
C23—N3—Ir1	91.7 (5)	C22—C21—H21	119.6
C25—N3—Ir1	138.1 (6)	C21—C22—N2	121.1 (9)
C2—C1—C6	117.2 (8)	C21—C22—H22	119.4
C2—C1—Ir1	128.2 (7)	N2—C22—H22	119.4
C6—C1—Ir1	114.6 (7)	O1—C23—N3	115.7 (7)
C3—C2—C1	121.9 (10)	O1—C23—C24	118.2 (8)
C3—C2—H2	119.1	N3—C23—C24	126.1 (9)
C1—C2—H2	119.1	O1—C23—Ir1	57.9 (4)
C2—C3—C4	119.1 (10)	N3—C23—Ir1	57.7 (4)
C2—C3—H3	120.5	C24—C23—Ir1	175.9 (7)
C4—C3—H3	120.5	C23—C24—H24A	109.5
C5—C4—C3	120.8 (10)	C23—C24—H24B	109.5
C5—C4—H4	119.6	H24A—C24—H24B	109.5
C3—C4—H4	119.6	C23—C24—H24C	109.5
C4—C5—C6	120.3 (10)	H24A—C24—H24C	109.5
C4—C5—H5	119.8	H24B—C24—H24C	109.5
C6—C5—H5	119.8	N3—C25—C26	118.6 (9)
C5—C6—C1	120.7 (9)	N3—C25—C30	123.7 (9)
C5—C6—C7	123.8 (9)	C26—C25—C30	117.7 (10)
C1—C6—C7	115.6 (8)	C27—C26—C25	120.8 (10)
N1—C7—C8	119.3 (9)	C27—C26—H26	119.6
N1—C7—C6	113.3 (8)	C25—C26—H26	119.6
C8—C7—C6	127.3 (9)	C28—C27—C26	120.3 (11)
C9—C8—C7	120.5 (10)	C28—C27—H27	119.9
C9—C8—H8	119.8	C26—C27—H27	119.9
C7—C8—H8	119.8	C29—C28—C27	120.6 (11)
C8—C9—C10	119.6 (10)	C29—C28—Cl1	118.7 (10)
C8—C9—H9	120.2	C27—C28—Cl1	120.7 (10)
C10—C9—H9	120.2	C28—C29—C30	121.0 (11)
C11—C10—C9	118.1 (10)	C28—C29—H29	119.5
C11—C10—H10	120.9	C30—C29—H29	119.5
C9—C10—H10	120.9	C25—C30—C29	119.5 (11)
N1—C11—C10	123.0 (9)	C25—C30—H30	120.2
N1—C11—H11	118.5	C29—C30—H30	120.2
C10—C11—H11	118.5		
			
C1—Ir1—O1—C23	31 (2)	C9—C10—C11—N1	−1.0 (15)
N2—Ir1—O1—C23	−174.5 (6)	C1—Ir1—C12—C13	−90.0 (8)
N1—Ir1—O1—C23	87.0 (5)	N2—Ir1—C12—C13	−176.8 (8)
C12—Ir1—O1—C23	−93.5 (5)	N1—Ir1—C12—C13	−106 (4)
N3—Ir1—O1—C23	−0.2 (5)	N3—Ir1—C12—C13	22.2 (8)
C1—Ir1—N1—C11	177.9 (7)	O1—Ir1—C12—C13	81.9 (8)
N2—Ir1—N1—C11	−95.7 (7)	C23—Ir1—C12—C13	52.7 (8)
C12—Ir1—N1—C11	−166 (4)	C1—Ir1—C12—C17	89.0 (6)
N3—Ir1—N1—C11	65.8 (7)	N2—Ir1—C12—C17	2.1 (6)
O1—Ir1—N1—C11	6.2 (7)	N1—Ir1—C12—C17	73 (4)
C23—Ir1—N1—C11	35.4 (7)	N3—Ir1—C12—C17	−158.9 (6)
C1—Ir1—N1—C7	−0.2 (6)	O1—Ir1—C12—C17	−99.2 (6)
N2—Ir1—N1—C7	86.2 (6)	C23—Ir1—C12—C17	−128.4 (6)
C12—Ir1—N1—C7	16 (4)	C17—C12—C13—C14	1.9 (14)
N3—Ir1—N1—C7	−112.3 (6)	Ir1—C12—C13—C14	−179.2 (7)
O1—Ir1—N1—C7	−171.9 (6)	C12—C13—C14—C15	−1.1 (16)
C23—Ir1—N1—C7	−142.7 (6)	C13—C14—C15—C16	−0.5 (17)
C1—Ir1—N2—C22	84.0 (8)	C14—C15—C16—C17	1.2 (17)
N1—Ir1—N2—C22	4.1 (8)	C13—C12—C17—C16	−1.1 (13)
C12—Ir1—N2—C22	−179.7 (8)	Ir1—C12—C17—C16	179.9 (7)
N3—Ir1—N2—C22	−106.4 (11)	C13—C12—C17—C18	178.5 (8)
O1—Ir1—N2—C22	−91.8 (8)	Ir1—C12—C17—C18	−0.5 (9)
C23—Ir1—N2—C22	−95.3 (8)	C15—C16—C17—C12	−0.4 (15)
C1—Ir1—N2—C18	−99.6 (6)	C15—C16—C17—C18	180.0 (9)
N1—Ir1—N2—C18	−179.5 (6)	C22—N2—C18—C19	0.6 (12)
C12—Ir1—N2—C18	−3.3 (6)	Ir1—N2—C18—C19	−176.3 (7)
N3—Ir1—N2—C18	69.9 (12)	C22—N2—C18—C17	−179.0 (8)
O1—Ir1—N2—C18	84.6 (6)	Ir1—N2—C18—C17	4.1 (10)
C23—Ir1—N2—C18	81.1 (7)	C12—C17—C18—C19	178.0 (8)
C1—Ir1—N3—C23	−174.4 (5)	C16—C17—C18—C19	−2.3 (15)
N2—Ir1—N3—C23	16.9 (12)	C12—C17—C18—N2	−2.4 (11)
N1—Ir1—N3—C23	−94.9 (5)	C16—C17—C18—N2	177.3 (9)
C12—Ir1—N3—C23	88.2 (5)	N2—C18—C19—C20	0.6 (14)
O1—Ir1—N3—C23	0.2 (5)	C17—C18—C19—C20	−179.8 (9)
C1—Ir1—N3—C25	6.3 (10)	C18—C19—C20—C21	−1.1 (16)
N2—Ir1—N3—C25	−162.4 (10)	C19—C20—C21—C22	0.3 (16)
N1—Ir1—N3—C25	85.8 (9)	C20—C21—C22—N2	1.0 (15)
C12—Ir1—N3—C25	−91.1 (9)	C18—N2—C22—C21	−1.4 (13)
O1—Ir1—N3—C25	−179.1 (10)	Ir1—N2—C22—C21	174.9 (7)
C23—Ir1—N3—C25	−179.3 (12)	Ir1—O1—C23—N3	0.3 (8)
N2—Ir1—C1—C2	79.6 (8)	Ir1—O1—C23—C24	−178.4 (8)
N1—Ir1—C1—C2	177.6 (8)	C25—N3—C23—O1	179.1 (8)
C12—Ir1—C1—C2	−1.3 (8)	Ir1—N3—C23—O1	−0.3 (8)
N3—Ir1—C1—C2	−96.6 (8)	C25—N3—C23—C24	−2.3 (15)
O1—Ir1—C1—C2	−125.3 (17)	Ir1—N3—C23—C24	178.3 (9)
C23—Ir1—C1—C2	−101.3 (9)	C25—N3—C23—Ir1	179.4 (11)
N2—Ir1—C1—C6	−99.0 (6)	C1—Ir1—C23—O1	−171.8 (6)
N1—Ir1—C1—C6	−1.0 (6)	N2—Ir1—C23—O1	7.1 (7)
C12—Ir1—C1—C6	−179.9 (6)	N1—Ir1—C23—O1	−94.7 (5)
N3—Ir1—C1—C6	84.8 (6)	C12—Ir1—C23—O1	86.8 (5)
O1—Ir1—C1—C6	56 (2)	N3—Ir1—C23—O1	179.7 (9)
C23—Ir1—C1—C6	80.1 (8)	C1—Ir1—C23—N3	8.5 (8)
C6—C1—C2—C3	−1.9 (14)	N2—Ir1—C23—N3	−172.6 (5)
Ir1—C1—C2—C3	179.6 (7)	N1—Ir1—C23—N3	85.6 (5)
C1—C2—C3—C4	0.5 (16)	C12—Ir1—C23—N3	−92.9 (5)
C2—C3—C4—C5	1.0 (16)	O1—Ir1—C23—N3	−179.7 (9)
C3—C4—C5—C6	−1.1 (16)	C1—Ir1—C23—C24	−152 (10)
C4—C5—C6—C1	−0.3 (14)	N2—Ir1—C23—C24	27 (10)
C4—C5—C6—C7	178.0 (9)	N1—Ir1—C23—C24	−75 (10)
C2—C1—C6—C5	1.7 (13)	C12—Ir1—C23—C24	106 (10)
Ir1—C1—C6—C5	−179.5 (7)	N3—Ir1—C23—C24	−161 (10)
C2—C1—C6—C7	−176.7 (8)	O1—Ir1—C23—C24	20 (10)
Ir1—C1—C6—C7	2.1 (10)	C23—N3—C25—C26	148.1 (10)
C11—N1—C7—C8	1.0 (13)	Ir1—N3—C25—C26	−32.7 (14)
Ir1—N1—C7—C8	179.3 (7)	C23—N3—C25—C30	−33.9 (15)
C11—N1—C7—C6	−176.8 (8)	Ir1—N3—C25—C30	145.3 (9)
Ir1—N1—C7—C6	1.4 (10)	N3—C25—C26—C27	−179.9 (10)
C5—C6—C7—N1	179.4 (8)	C30—C25—C26—C27	2.0 (17)
C1—C6—C7—N1	−2.3 (11)	C25—C26—C27—C28	0.2 (19)
C5—C6—C7—C8	1.7 (15)	C26—C27—C28—C29	−1 (2)
C1—C6—C7—C8	−179.9 (9)	C26—C27—C28—Cl1	179.2 (10)
N1—C7—C8—C9	−1.0 (15)	C27—C28—C29—C30	1 (2)
C6—C7—C8—C9	176.6 (10)	Cl1—C28—C29—C30	179.9 (9)
C7—C8—C9—C10	−0.1 (17)	N3—C25—C30—C29	179.1 (10)
C8—C9—C10—C11	1.1 (16)	C26—C25—C30—C29	−2.9 (16)
C7—N1—C11—C10	−0.1 (14)	C28—C29—C30—C25	1.7 (18)
Ir1—N1—C11—C10	−178.1 (7)		
